# Echocardiographic evaluation of left ventricular filling pressures in patients with pulmonary hypertension

**DOI:** 10.1007/s10554-019-01528-6

**Published:** 2019-01-21

**Authors:** Hong Ran, Matthias Schneider, Anna Maria Pistritto, Christian Gerges, Houtan Heidari, Thomas Binder, Irene Lang, Georg Goliasch

**Affiliations:** 1 Department of Internal Medicine II, Medical University of Vienna, Waehringer Guertel 18-20, 1090 Vienna, Austria; 2 Department of Echocardiography, Nanjing First Hospital, Nanjing Medical University, Nanjing, China; 3 Division of Cardiology, Emergency Department, San Paolo Hospital, Savona, Italy

**Keywords:** Echocardiography, Diastolic function, Pulmonary hypertension, Elevated left ventricular filling pressures

## Abstract

Echocardiographic assessment of diastolic dysfunction depends on surrogate parameters. In recent years, guideline committees attempted to combine these parameters to diagnostic flowcharts allowing for correct classification of left ventricular filling pressures (LVFP). The value of these diagnostic tools is limited if the applied surrogate parameters are elevated due to other reasons as is the case with maximal tricuspid regurgitation velocity. We aimed to compare the accuracy of the 2009 and the 2016 guideline recommendations in patients with pulmonary hypertension (PH). We included 101 consecutive patients who underwent right heart catheterization and transthoracic echocardiography for suspicion of PH. For the final analysis, only patients with PH were considered. The 2009 and 2016 recommendations for the assessment of diastolic function by echocardiography were applied on each patient. A total of 63 PH patients were included in the final analysis, 43% had elevated LVFP. By using the 2009 recommendations, sensitivity for correct classification of diastolic dysfunction was 67%, specificity was 82%, area under the curve (AUC) was 0.74. By using the 2016 recommendations, sensitivity for correct classification of diastolic dysfunction was 84%, specificity was 80%, AUC was 0.82. In ROC comparison, the AUC for the 2016 recommendations with 0.82 was significantly better compared to the AUC of 0.74 for the 2009 recommendations (p = 0.04). Our study demonstrates that the 2016 recommendations for echocardiographic evaluation of diastolic function are superior to the 2009 recommendations in estimating left ventricular filling pressures in patients with PH.

**Electronic supplementary material:**

The online version of this article (10.1007/s10554-019-01528-6) contains supplementary material, which is available to authorized users.

## Background

Pulmonary hypertension (PH) is a disease with severe morbidity and mortality. Left heart disease (LHD) is the most common underlying condition in PH [1, 2]. In PH–LHD, right ventricular (RV) failure can develop in the course of the disease. When evaluated by echocardiography, especially in the case of heart failure with preserved ejection fraction (HFpEF), detection of PH and RV failure can lead to the false assumption that pre-capillary PH is the underlying disease. At the same time, post-capillary PH with RV dysfunction resembles a high-risk population and must be followed closely [3–5].

Elevated left ventricular filling pressure (LVFP) can be assessed by echocardiography. Several parameters have been evaluated for the echocardiographic diagnosis of left ventricular (LV) diastolic dysfunction. In 2009, a series of parameters were combined to a diagnostic algorithm which were published as recommendations for the evaluation of LV diastolic function [6]. In 2016, an updated version was published, suggesting a more simplified approach [7]. In the Euro-Filling Study, both recommendations were compared and the diagnostic accuracy of both approaches was studied. The analysis revealed superiority of the 2016 recommendations when compared with the 2009 approach [8]. Both recommendations primarily rely on the secondary findings left atrial (LA) dilatation, maximal tricuspid regurgitation velocity (TRvmax), and the Doppler criteria E/A, E′ and E/e′ (Fig. 3). TRvmax is a surrogate for elevated systolic pulmonary artery pressure and is thus elevated in patients with pulmonary hypertension due to any cause. The fact that TRvmax plays a major role in the algorithms for the diagnosis of diastolic dysfunction can lead to false interpretation of diastolic function in patients with PH of other causes than LHD. This must result in false-positives in patients with isolated pre-capillary pulmonary hypertension. In this study, we aimed to investigate both the 2009 and the 2016 recommendations to determine which is more reliable to diagnose elevated LVFP in patients with PH.

## Methods

### Study population

We included all adult patients with clinically indicated right heart catheterization (RHC) between July 2015 and July 2016. For the final analysis, only patients with PH (invasively measured mean pulmonary artery pressure (mPAP) ≥ 25 mmHg (1)) were included. The study was conducted in accordance with the amended Declaration of Helsinki. The ethic committee of the Medical University of Vienna approved the conduct of the study (EK# 2012/2014). All patients gave written informed consent before enrollment. Exclusion criteria were patients < 18 years of age.

### Echocardiographic examination

Standard transthoracic echocardiograms (2D, Doppler) were performed in all enrolled patients shortly before invasive hemodynamic assessment with echocardiography systems equipped with 3.5 MHz transducers (Vivid E9, Vivid S70; General Electric Healthcare) according to the recommendations and guidelines by the American Society of Echocardiography and the European Association of Cardiovascular Imaging [9].

In an apical 4-chamber and 2-chamber view, left atrial volume was measured by biplane Simpson method. The volume was indexed for body surface area. In an apical four-chamber view, pulsed wave Doppler mitral in-flow was recorded. Trans-mitral E wave and A wave peak velocities were measured, E/A ratio was calculated. In an apical 4-chamber-view, e′ velocity was measured both at the basal and the lateral mitral annulus. Both e′ values were averaged, E/e′ ratios were measured for average, for lateral, and for septal e′. Peak tricuspid regurgitation velocity was also measured.

In addition to the standard measurements, regional peak atrial longitudinal strain was analyzed in the basal segment of the interatrial septum of the left atrium (LA). To ensure for correct echocardiographic classification, diastolic dysfunction was independently assessed by the same two observers (HR, MS) in all patients.

The 2009 recommendations for the evaluation of left ventricular diastolic function by echocardiography suggest two different approaches depending on left ventricular ejection fraction. In our study, all included patients had normal LVEF. Thus, the pathway for estimation of filling pressures in patients with normal ejection fraction was used for this analysis.

The 2016 recommendations for the evaluation of left ventricular diastolic function by echocardiography suggests two approaches. One for patients with normal LVEF, and one for patients with depressed LVEF and patients with myocardial disease. In our analysis, we analyzed both flowcharts.

### Hemodynamic assessment

Invasive hemodynamic assessment was performed in all study participants. Mean time difference between echocardiography and right heart catheter was 0.62 days. Hemodynamic measurements were performed using a 7F Swan-Ganz catheter (Edwards Lifesciences GmbH, Austria) via a femoral access. Pressures were documented as average of eight measurements over eight consecutive heart cycles using CathCorLX (Siemens AG, Berlin and Munich, Germany). In addition to mean pulmonary arterial wedge pressure (mPAWP), the systolic, diastolic, and mean (mPAP) pulmonary artery (PA) pressures were measured. In a subgroup of patients left ventricular end-diastolic pressure (LVEDP) was measured via left heart catheterization where clinically indicated (suspicion for coronary artery disease, pre-operative assessment of left heart valve disease). Left heart catheterization was performed directly after right heart catheterization.

We therefore defined LVFP as elevated if mPAWP > 12 mmHg if available, in the remaining patients if LVEDP > 16 mmHg [10, 11].

### Statistical analysis

Continuous variables are given as mean +/− standard deviation (SD). Pearson correlation coefficients were calculated to compare invasive with echo measurements. Specificity and sensitivity were calculated for the different diagnostic approaches. Area under the curve (AUC) of the ROC curve was calculated to examine the power of the different measurements. Intraclass correlation coefficients were used to report interobserver and intraobserver variability for LA volume, TR velocity, and e′ in ten randomly selected patients. Differences between two groups were analyzed by T-test analysis. A p value ≤ 0.05 was considered statistically significant. SPSS Version 24 (IBM SPSS, USA) was used for all analyses.

## Results

### Patient characteristics

A total of 63 patients were included in the final analysis, 35 patients (56%) were female. Mean age was 68 years (range 21–91). Left ventricular systolic function was normal in all patients. Twenty-eight (44%) had PH-LHD, 20 (32%) had chronic-thromboembolic PH, nine (14%) had pulmonary arterial hypertension. Complete patient characteristics are shown in Table 1.

**Table 1:** Baseline characteristics of total study population (n = 63)

Baseline characteristics	
Age, years (mean, SD)	68 (15)
Female sex, n (%)	35 (56)
Coronary artery disease, n (%)	18 (29)
Diabetes mellitus, n (%)	12 (19)
Arterial hypertension, n (%)	55 (87)
Systolic blood pressure, mmHg (SD)	143 (26)
Diastolic blood pressure, mmHg (SD)	85 (14)
Hyperlipidemia, n (%)	32 (51)
History of smoking, n (%)	20 (32)
Clinical classification of pulmonary hypertension	
Class 1: Pulmonary arterial hypertension, n (%)	9 (14)
Class 2: PH due to left heart disease, n (%)	28 (44)
Class 3: PH due to lung disease, n (%)	4 (6)
Class 4: Chronic thromboembolic PH, n (%)	20 (32)
Class 5: PH with unclear and/or multifactorial mechanism, n (%)	2 (3)
Laboratory results	
proBNP, pg/ml (SD)	2849 (5250)
Creatinin, mg/dl (SD)	1.0 (0.37)
Echocardiographic data	
LVEDVi, ml (SD)	42 (14)
LVESV, ml (SD)	26 (11)
LVEF biplane Simpson method, % (SD)	68 (7)
LAVi, ml/m² (SD)	39 (22)
Mitral E wave, m/s (SD)	0.8 (0.4)
E/A ratio (SD)	1.4 (1)
Septal e′, cm/s (SD)	0.07 (0.02)
Lateral e′, cm/s (SD)	0.11 (0.04)
E/e′ septal (SD)	13 (9)
E/e′ lateral (SD)	8 (4)
E/e′, average (SD)	10 (5)
TR velocity, m/s (SD)	3.8 (0.77)
sPAP, mmHg (SD)	69 (23)
TAPSE, mm (SD)	17 (4)
RVF reduced, n (%)	29 (46)
Valve disease	
TR ≥ moderate, n (%)	27 (43)
MR ≥ 2, n (%)	11 (17)
AR ≥ 2, n (%)	5 (8)
Invasive data	
mPAP, mmHg (SD)	43 (13)
LVEDP, mmHg (SD)	15 (7)
mPAWP, mmHg (SD)	14 (7)

*PH* pulmonary hypertension, *LVEDVi* left ventricular end-diastolic volume index, *LVESV* left ventricular end-systolic volume, *LVEF* left ventricular ejection fraction, *LAVi* left atrial volume index, *TR* tricuspid regurgitation, *sPAP* systolic pulmonary artery pressure, *TAPSE* tricuspid annular plane systolic excursion, *RVF* right ventricular function, *MR* mitral regurgitation, *AR* mitral regurgitation, *mPAP* mean pulmonary artery pressure, *LVEDP* left ventricular end-diastolic pressure, *mPAWP* mean pulmonary arterial wedge pressure

### Echocardiographic examination

At least moderate tricuspid regurgitation (TR) was present in 27 patients (43%), at least moderate mitral regurgitation was present in 11 patients (17%). The RV was dilated in 31 patients (49%), and RV function was reduced in 29 patients (46%) with a mean TAPSE of 17 mm (SD 4) and a mean S′ of 0.109 m/s (SD 0.03). Mean TRvmax was 3.8 m/s (SD 0.77), mean estimated systolic pulmonary artery pressure (sPAP) was 69 mmHg (SD 23). Mean E/A ratio was 1.4, mean septal E/e′ was 13, mean lateral E/e′ 8, and mean averaged E/e′ was 10 (for complete echocardiographic data see Table 1).

Intraclass correlation for interobserver variability for LA volume, TR velocity, and lateral e′ was 0.95 (95% CI 0.82–0.99, p < 0.001), 0.99 (95% CI 0.98–0.99, p < 0.001), and 0.99 (95% CI 0.95–0.99, p < 0.001) respectively, intraobserver variability was 0.92 (95% CI 0.68–0.98, p < 0.001), 0.99 (95% CI 0.97–0.99, p < 0.001), and 0.99 (95% CI 0.97–0.99, p < 0.001) respectively.

### Invasive hemodynamics

mPAWP was measured in 60 patients (95%), LVEDP was measured in 46 patients (73%). At least one of the two parameters was measured in each patient included in the final analysis. All patients had PH (mPAP ≥ 25 mmHg). A total of 27 patients (43%) had elevated LVFP (PH–LHD). Invasive hemodynamic data are listed in Table 1.

### Correlation of echo parameters with invasive data

There was significant correlation with mPAWP for E/A (r = 0.55, p < 0.001), E/e′ (r = 0.55, p < 0.001), TRvmax (r = − 0.36, p = 0.005), and LA volume index (r = 0.66, p < 0.001), Fig. 2. There was significant correlation with LVEDP for E/e′ (r = 0.49, p = 0.002), TRvmax (r = − 0.37, p = 0.015), and LA volume index (r = 0.47, p = 0.001).

Area under the curve for correct determination of mPAWP > 12 mmHg was 0.86, 0.81, 0.7, and 0.3 for LA volume index, E/e′ lateral, E/e′ septal, and TRvmax, respectively.

**Table 2:** Sensitivity, Specificity, positive and negative predictive value, accuracy, for the 2009 and 2016 (figure B) recommendations

	2009 Recommendations	2016 Recommendations
Sensitivity, % (95% CI)	67 (47–83)	84 (66–95)
Specificity, % (95% CI)	82 (63–94)	80 (61–92)
Positive predictive value, % (95% CI)	80 (63–90)	81 (68–90)
Negative predictive value, % (95% CI)	70 (57–80)	83 (68–92)
AUC, % (95% CI)	74* (61–85)	82* (70–91)

* Indicates a statistically significant difference with a *p* value of ≤ 0.05

*AUC* area under the curve

### Accuracy of echocardiography using 2009 and 2016 recommendations

**Fig. 1:**
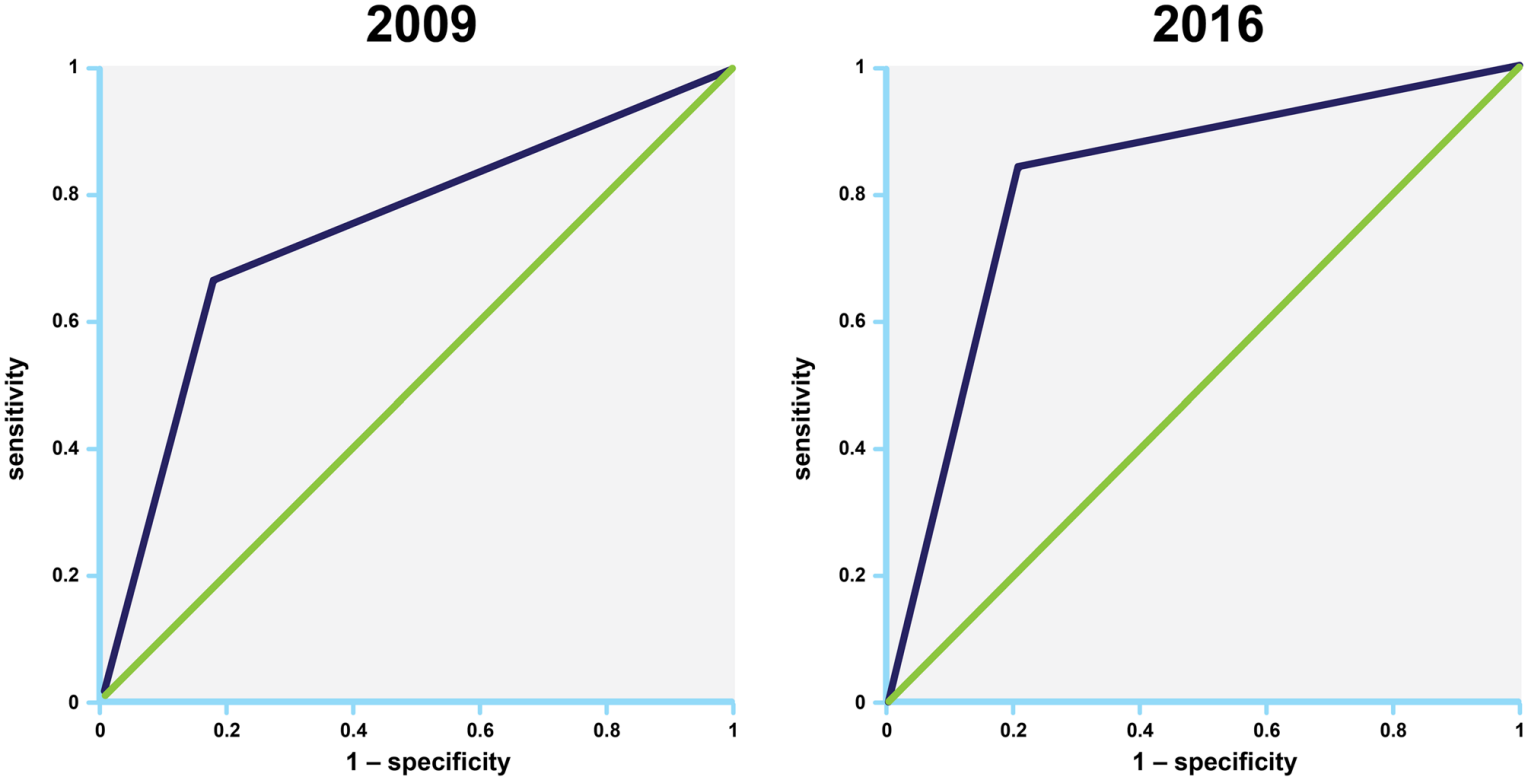
Comparison of receiver operating curves (ROC) between the 2009 (left) and 2016 (right) recommendations for the assessment of diastolic function. In ROC comparison, the area under the curve (AUC) for the 2016 recommendations with 0.82 was significantly better compared to the AUC of 0.74 for the 2009 recommendations (p = 0.0427)

By applying the 2009 recommendations, LV filling pressures were estimated as normal in 31 patients (49%), elevated in 27 patients (43%), and indeterminate in five patients (8%). Sensitivity for correct classification of diastolic dysfunction was 67%, specificity was 82%, area under the curve (AUC) was 74% (for complete data see Table 2). By applying the 2016 recommendations for patients with normal LVEF, 22 cases were indeterminate. LV filling pressures were estimated as normal in 16 patients (25%), and as elevated in 20 patients (32%). By applying the 2016 recommendations for patients with myocardial disease, LV filling pressures were estimated as normal in 29 patients (46%), and elevated in 33 patients (52%). One patient was in atrial fibrillation and did not have an E/A signal. Sensitivity for correct classification of diastolic dysfunction was 84%, specificity was 80%, AUC was 82%. In ROC comparison, the AUC for the 2016 recommendations with 0.82 was significantly better compared to the AUC of 0.74 for the 2009 recommendations (p = 0.0427), Fig. 1.

**Table 3:** Comparison of those patients with elevated and with normal left ventricular filling pressures (LVFP)

	Elevated LVFP (27 patients)	Normal LVFP (36 patients)	p-Value
Baseline characteristics			
Age, mean (SD)	73.5 (11)	61.5 (17)	0.002*
proBNP pg/ml (SD)	3065 (4113)	2711 (6365)	0.795
Creatinin, mg/dl (SD)	1.11 (0.39)	0.97 (0.34)	0.124
Pulmonary hypertension			
mPAP (right heart cath.), mmHg (SD)	40 (12.5)	45.5 (13.6)	0.110
Maximal TR velocity, m/s (SD)	3.5 (0.71)	4.1 (0.72)	0.002*
Diastolic dysfunction parameters			
PALS basal interatrial septum, % (SD)	4.8 (5.9)	8.3 (7.6)	0.05*
LA volume index, ml/m2 (SD)	51 (24)	27 (10)	< 0.001*
E/e′ average, ratio (SD)	12.6 (5.8)	7.4 (3.4)	< 0.001*
E/e′ septal, ratio (SD)	12.8 (7)	14.7 (11)	0.517
E/e′ lateral, ratio (SD)	10.1 (5)	5.6 (2.5)	< 0.001*
Right ventricular size and function			
TAPSE, mm (SD)	16.9 (4.6)	17.3 (4)	0.72
S′, m/s (SD)	0.11 (0.03)	0.11 (0.02)	0.914
RV dilated, %	46	74	0.005*
Left ventricular function			
Biplane ejection fraction, % (SD)	66.7 (6)	68.5 (7)	0.284
LV global longitudinal strain, % (SD)	− 14.4 (4.8)	− 15.4 (3.8)	0.363

* Indicates a statistically significant difference with a *p* value of ≤ 0.05

*mPAP* mean pulmonary artery pressure, *PALS* peak atrial longitudinal strain, *LA* left atrium, *TAPSE* tricuspid annular plane systolic excursion, *RV* right ventricle, *LV* left ventricle

### Comparison of patients with normal and with elevated LVFP

In the subgroup of patients with elevated LVFP, patients were older, and less patients were female. TRvmax was significantly lower, LA volume index was larger. There was no difference in left ventricular global longitudinal strain (GLS) and LVEF. Parameters for RV function were the same in both groups but the right ventricle was dilated significantly more often in those with normal LVFP. mPAP measured in RHC showed no significant difference in the two groups. E/e′ on average was significantly more elevated in the LVFP group, which was mediated by the lateral measurements, septal measurements did not differ. Regional peak atrial longitudinal strain at the level of the basal interatrial septum was significantly reduced in the elevated LVFP group (for complete data see Table 3).

## Discussion

**Fig. 2:**
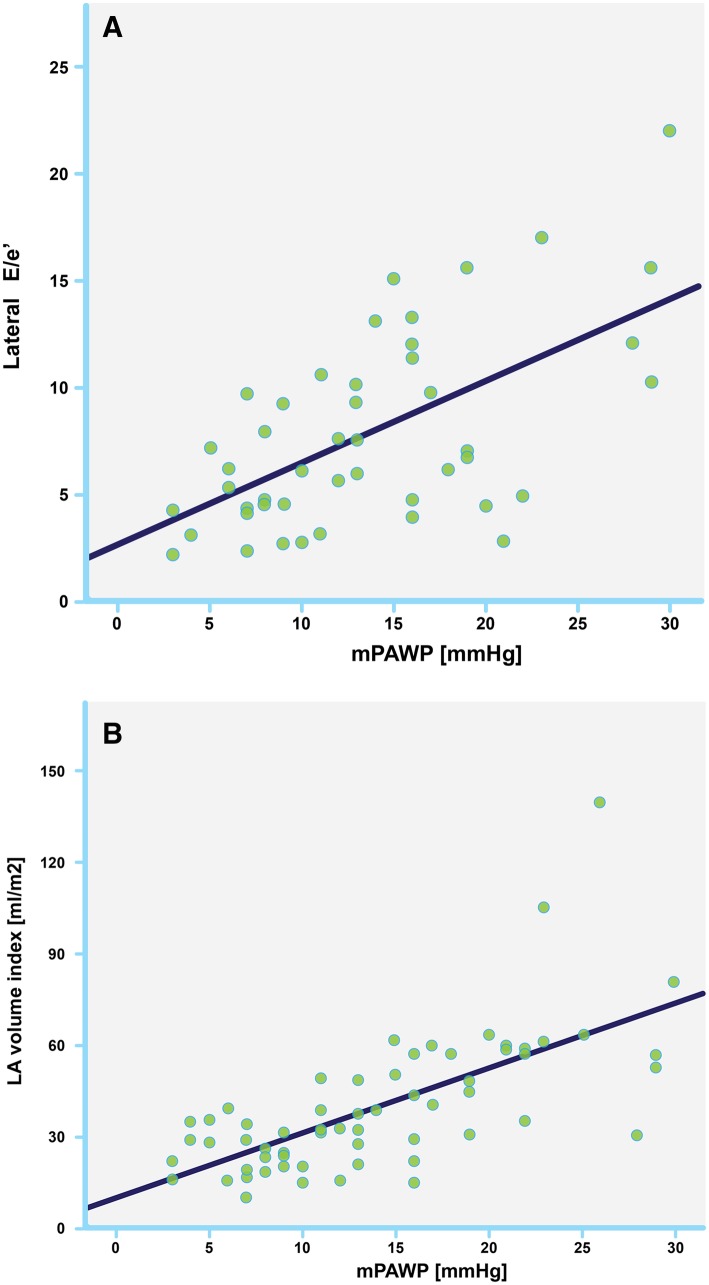
Scatter plots showing correlation with mean pulmonary arterial wedge pressure (mPAWP) for E/e′ (**a**, r = 0.55, p < 0.001), and left atrial volume index (**b**, r = 0.66, p < 0.001)

**Fig. 3:**
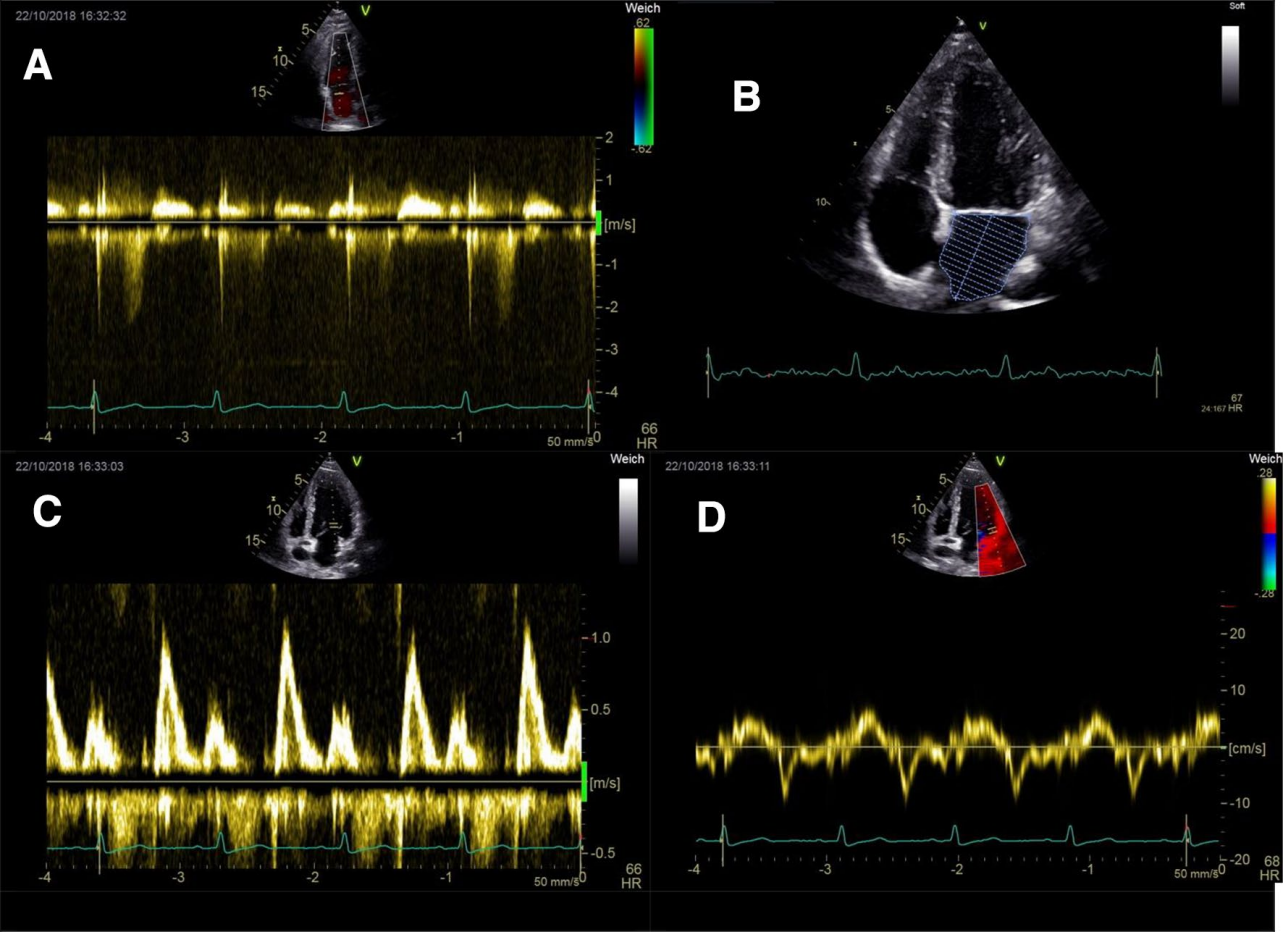
Echocardiographic surrogate parameters allowing for the assessment of left ventricular filling pressures. **a** Maximal tricuspid regurgitation velocity (TRvmax). **b** Volume assessment of the left atrium. **c** PW-Doppler signal of mitral inflow, E-wave and A-wave. **d** Tissue Doppler imaging of lateral mitral valve annulus, e′. *PW* pulsed wave

Echocardiographic assessment of diastolic dysfunction depends on surrogate parameters. In recent years, guideline committees attempted to combine these parameters to diagnostic flowcharts allowing for correct classification of LVFP. The value of these diagnostic tools is limited if the applied surrogate parameters are elevated due to other conditions. This especially applies for TRvmax which plays a prominent role in both the 2009 and the 2016 recommendations. In our work, we could show that the 2016 diagnostic approach is superior to the 2009 approach in patients with elevated TRvmax due to PH of any cause (Figs. 2, 3).

In patients with echocardiographic signs of PH it is of great clinical importance to understand the underlying disease and to differentiate between post-capillary, pre-capillary, and combined pre- and post-capillary pulmonary hypertension. Thus, invasive hemodynamic assessment is mandatory in all patients with suspicion for PH. However, by its invasive nature cardiac catheterization is associated with potential complications. Therefore, continuous improvements in imaging techniques with increasing accuracy are needed. Nevertheless, as of today, echocardiography cannot sufficiently differentiate between pre- and post-capillary PH, which has been demonstrated recently by D’Alto et al. with a positive predictive value (PPV) of 68% and a negative predictive value (NPV) of 77% [12].

The main disadvantage of the 2009 recommendations is the number of patients who have to be classified as indeterminate due to inconclusive results of the measured parameters. The 2016 recommendations allowed for classification of 98% of the patients in our study. Through this classification a PPV of 81% and a NPV of 83% could be reached predicting the presence or absence of elevated LVFP. In ROC comparison, the AUC for the 2016 recommendations with 0.82 was significantly better compared to the AUC of 0.74 for the 2009 recommendations (p = 0.0427).

All echocardiographic parameters estimating LVFP correlated significantly with mPAWP. Patients with elevated LVFP had significantly larger LA volumes and lateral E/e′ was significantly higher. Septal E/e′ did not show a significant difference between the two groups. It can be assumed, that the abnormal septal movement in patients with RV volume and pressure overload could falsely influence e′. Thus, septal E/e′ needs to be used with specific caution in PH patients.

Multiple adaptions have led to a higher diagnostic accuracy of the 2016 recommendations in comparison with those from 2009. While E/e′ played a central role in distinguishing between the diagnosis of high versus normal LVFP in 2009, the 2016 recommendations only evaluate E/e′ as one of several parameters. This can correct for false-low E/e′ as discussed above. While the 2009 recommendations suggest to calculate systolic pulmonary artery pressure with the many pitfalls known to be associated with this estimation, the 2016 recommendations rely on TRvmax. This reduces the chance for wrong classifications. The 2009 recommendations suggested several further indirect echocardiographic measurements for elevated LVFP which did not improve the diagnostic accuracy but complicated the assessment. The 2016 recommendations succeeded in simplifying the diagnostic approach leading to superior diagnostic accuracy at the same time. However, there are still significant numbers of wrong classifications even with the 2016 recommendations. Future research should further aim to correctly classify PH patients into pre- and post-capillary etiology by echocardiography.

The new parameter peak atrial longitudinal strain (PALS) has previously been established as excellent parameter identifying elevated LVFP [13, 14]. While there is a need for careful imaging of the entire LA to measure global LA strain, regional strain of the basal interatrial septum can be measured reliably in almost every patient. We evaluated regional PALS of the basal interatrial septum in our cohort. Absolute values were extremely low, possibly due to high right atrial pressure because of significant pulmonary hypertension. Nevertheless, it significantly distinguished between normal and elevated LVFP in our cohort. Future studies should focus on this parameter in the evaluation of LVFP.

## Limitations

The study population was small with only 63 patients. However, considering the complete hemodynamic and echocardiographic assessment in this patient collective with significant PH, the number nevertheless allows for important insights regarding diagnostic accuracy. Our data reflects the experience of a single tertiary care center. However, the potential advantages of a single-center approach are the enrolment of a homogenous patient population, the adherence to a consistent clinical routine, and a consistent quality of imaging procedures and RHC.

## Conclusion

With a PPV of 81% and a NPV of 83%, the 2016 recommendations on the assessment of diastolic function are superior to the 2009 recommendations in the subgroup of patients with concomitant pulmonary hypertension. Septal E/e′ should be considered with caution in patients with severe right ventricular volume and/or pressure overload due to abnormal septal movement. Further studies should focus on new parameters such as regional and global peak atrial longitudinal strain, which can be obtained easily and reliably in almost all patients.

## Electronic supplementary material

Below is the link to the electronic supplementary material.

Supplementary material 1 (DOCX 210 KB)

Supplementary material 2 (DOCX 194 KB)
