# Supplementary material for: Echocardiographic evaluation of left ventricular filling pressures in patients with pulmonary hypertension
Source: Int J Cardiovasc Imaging. 2019 Jan 21;35(5):861–8. doi: 10.1007/s10554-019-01528-6 (PMC6486531; doi:10.1007/s10554-019-01528-6)
Supplement: Supplementary file 1 — Supplementary material 1 (DOCX 210 KB) [file 10554_2019_1528_MOESM1_ESM.docx]

Supplementary material 1: 2009 recommendations for the assessment of diastolic function in patients with normal left ventricular ejection fraction, adapted from ASE recommendations. LA = left atrium; sPAP = systolic pulmonary artery pressure; Ar-A = time difference between the duration of pulmonary vein backward flow during atrial contraction and the duration of the A wave of the mitral inflow pulsed-wave Doppler signal; IVRT = isovolumetric relaxation time.

***
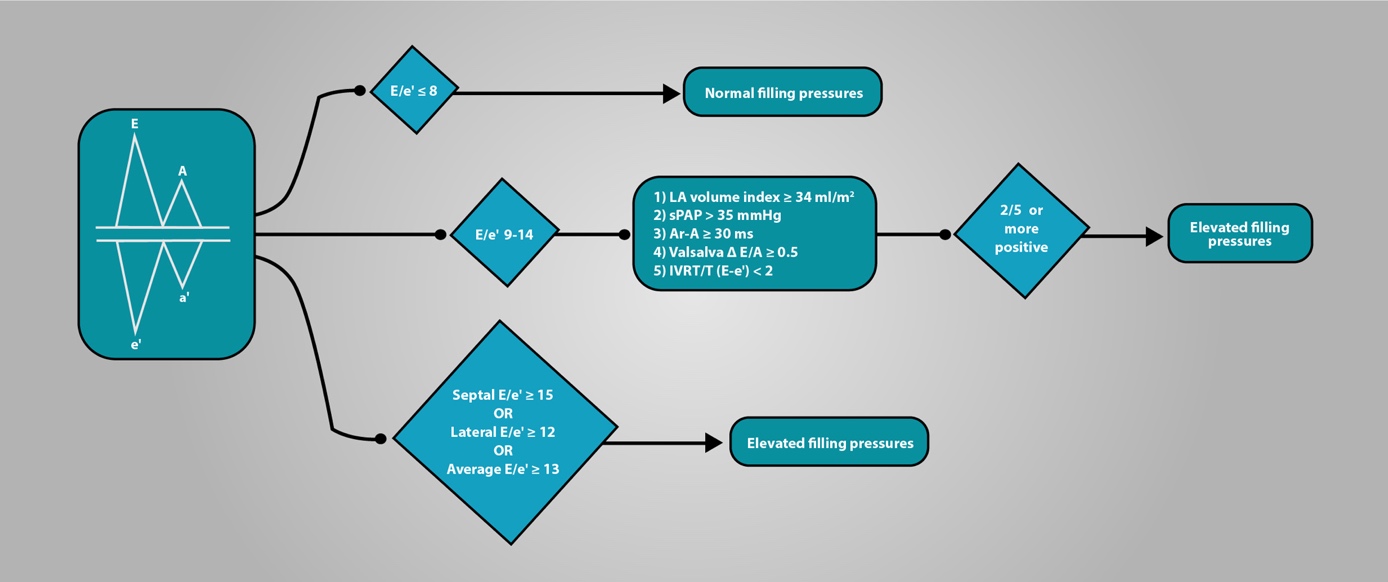
***
