# Supplementary material for: Echocardiographic evaluation of left ventricular filling pressures in patients with pulmonary hypertension
Source: Int J Cardiovasc Imaging. 2019 Jan 21;35(5):861–8. doi: 10.1007/s10554-019-01528-6 (PMC6486531; doi:10.1007/s10554-019-01528-6)
Supplement: Supplementary file 2 — Supplementary material 2 (DOCX 194 KB) [file 10554_2019_1528_MOESM2_ESM.docx]

Supplementary material 2: 2016 recommendations for the assessment of diastolic function in patients with depressed left ventricular ejection fraction (LVEF) or in patients with myocardial disease and normal LVEF, adapted from ASE/EASCVI recommendations. TR = tricuspid regurgitation; LA = left atrium.

***
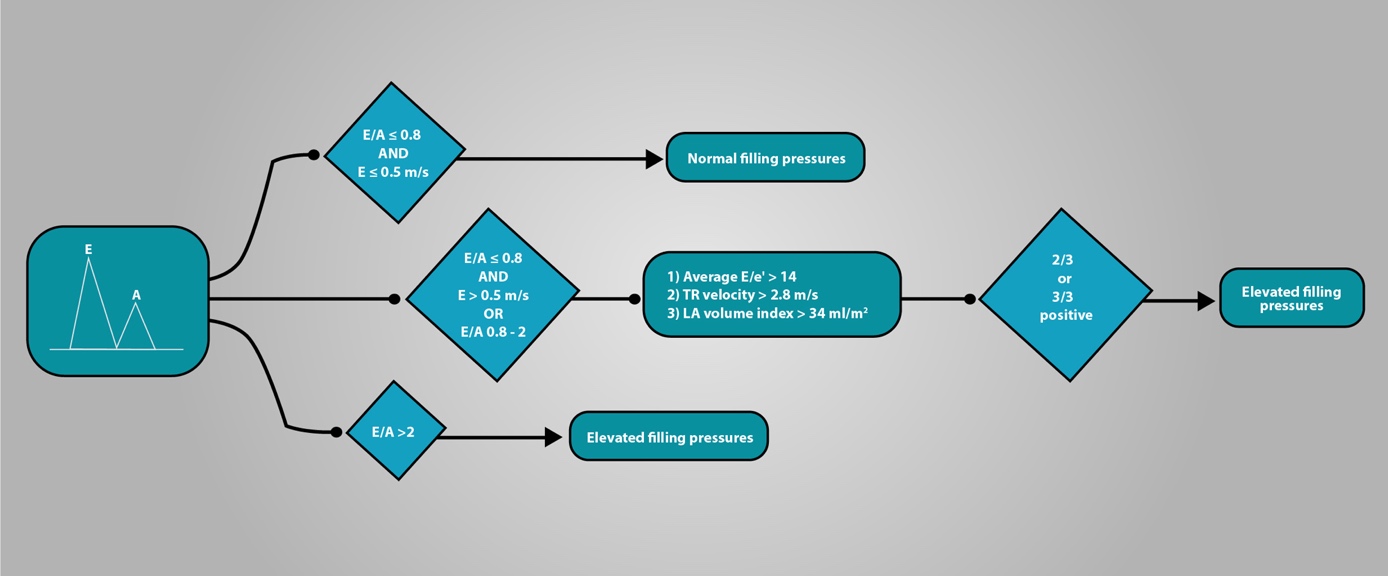
***
